# Exploring the relationship between health concerns and high‐risk behaviours in Medical Sciences' students

**DOI:** 10.1002/nop2.596

**Published:** 2020-08-20

**Authors:** Hoda Arabi‐Mianrood, Zeinab Hamzehgardeshi, Shayesteh Jahanfar, Mahmood Moosazadeh, Elham Khoori, Zohreh Shahhosseini

**Affiliations:** ^1^ Department of Reproductive Health and Midwifery Student Research Committee Mazandaran University of Medical Sciences Sari Iran; ^2^ Department of Reproductive Health and Midwifery Sexual and Reproductive Health Research Center Mazandaran University of Medical Sciences Sari Iran; ^3^ Ph.D in Reproductive Epidemiology Department of Community Health Central Michigan University Mount Pleasant MI USA; ^4^ Department of Epidemiology Health Sciences Research Center Addiction Institute Mazandaran University of Medical Sciences Sari Iran; ^5^ Department of Midwifery, Counseling and Reproductive Health Research Center Golestan University of Medical Sciences Gorgan Iran; ^6^ Department of Reproductive Health and Midwifery Ph.D in Reproductive Health, Sexual and Reproductive Health Research Center Mazandaran University of Medical Sciences Sari Iran

**Keywords:** health concerns, high‐risk behaviour, medical students, risk taking, university students, youth

## Abstract

**Aim:**

The need to cope with life concerns may drive an individual to resort to high‐risk behaviours. This study aimed to determine the relationship between health concerns and high‐risk behaviours.

**Design:**

A cross‐sectional study.

**Methods:**

We sampled 926 Medical Sciences' students from the North of Iran from September–December 2017 using the stratified sampling method.

**Results:**

The most and less common high‐risk behaviour was physical inactivity (97.5%) and high‐risk sexual behaviour (15.7%), respectively. In multivariate logistic regression analysis, participants' concerns about human sexuality (AOR: 1.39; CI: 1.22, 1.57), injury prevention and control (AOR: 1.12; CI: 1.01, 1.20), nutrition (AOR: 1.13; CI: 1.02, 1.26) and emotional health (AOR: 1.08; CI: 1.02, 1.15) increased the odds of risky behaviours. Among Medical Sciences' students, health concerns are linked with risky behaviours. The result of this study can be used to develop relevant interventions targeting mental health to reduce risky behaviour among youth.

## 1 INTRODUCTION

High‐risk behaviours among youth have grown into one of the leading and most widespread concerns of society despite attempts over the past three decades to address these problems (Duell et al., 2018; Hayashi et al., 2018; Stormshak et al., 2019). The literature elucidates 71% of the causes of death among 10–24 years old, which can lead to chronic diseases in youth (Saffier et al., 2017; West, 2017). Intentional and unintentional injuries, tobacco use, alcohol and drug use, high‐risk sexual behaviours, unhealthy dietary behaviours and physical inactivity are some examples of such behaviours (Ahmadi‐Montecalvo et al., 2019; Sorush et al., 2018).

A cross‐sectional study among 977 Iranian university students showed that during the last year alone, Hookah use (16.1%) and cigarette smoking (10%) had increased dramatically among youth. The frequency of alcohol and illicit drug use in the same group during the lifetime was found to be 11.9% and 8.8%, respectively (Afrashteh, Ghaem, Abbasi‐Ghahramanloo, & Tabatabaee, 2017). Although the prevalence of high‐risk sexual behaviours is controversial in the Iranian context, some studies reported extramarital sexual relationships among Iranian youth to be about 8%–41% (Mahmoodi et al., 2019; Rahmati‐Najarkolaei & Kamalikhah, 2014; Vakilian, Mousavi, & Keramat, 2014).

High‐risk conduct may appeal to individuals as a means of coping with concerns in their life (Kim, Kim, & Park, 2020; Noel et al., 2013; Soleimani et al., 2017). This issue highlights the need to examine the association between health concerns and high‐risk behaviours, as it can improve our understanding of the stimulants of risky conduct (Baheiraei, Khoori, Ahmadi, Rahimi Foroushani, & Ghofranipour, 2014; Teychenne et al., 2015). Although high‐risk behaviours are relatively common in the general population with psychological distress (Harakeh, Engels, Monshouwer, & Hanssen, 2010; Murphy et al., 2001; Tavolacci et al., 2013), less is known about the association between psychological distress and high‐risk behaviour among Medical Sciences' students.

There are several reasons why this issue should be given special attention in university students. According to the existing literature, emerging adults (people between 18–25 years of age), who are represented widely by university students, are believed to have higher tendency to adopt unhealthy behaviours than the other age groups (Laska et al., 2009; Tavolacci et al., 2013). Also, it is stated that following entering to the university and the more freedom from day‐to‐day parental control, the more participation in the peer groups, the more being in diverse social situations, the university students' worries and distresses increase and predispose them to high‐risk behaviours (Basic & Erdelez, 2015; Darling, McWey, Howard, & Olmstead, 2007; Griggs & Crawford, 2019). Finally, it showed that university students experiencing multiple forms of distress simultaneously as a result of multifactorial changes like the transition from leaving home and high school and adjusting to student life (Dyrbye et al., 2011; Hope & Henderson, 2014; Reis et al., 2019). Although it is anticipated that Medical Sciences' students adopt healthier practices as a result of better information concerning health issues (Chourdakis, Tzellos, Papazisis, Toulis, & Kouvelas, 2010), the literature is controversial (Arroyo et al., 2004; Yahia et al., 2008). Also, according to existing studies, medical students are more concerned due to reasons such as perfectionism and some aspects of medical education that are aimed to graduate knowledgeable, skilful and professional physicians. Studies suggest that mental health worsens after students begin medical school and remain poor throughout the training which consequently can predispose them to risky behaviours (Dyrbye et al., 2005; Jönsson & Ojehagen, 2006). So, the research in this group has some implication for health policy makers. Investigating the related factors to risky behaviours early in life may help decrease the burden of non‐communicable diseases in adult life and thus decrease the pressure on society and the healthcare system. Therefore, this study was implemented to assess the relationship between health concerns and high‐risk behaviours in the students of Mazandaran University of Medical Sciences (MAZUMS).

## 2 METHODS

### 2.1 Study setting and data collection

This cross‐sectional study was conducted at MAZUMS located in Mazandaran province, North of Iran, from September–December 2017. Initially, in this three‐stage stratified proportional to size sampling method, the sample size of each school of MAZUMS (Medicine, Dentistry, Pharmacy, Public Health, Nursing, and Allied Medical Sciences), as main strata, was obtained by dividing the total number of the students in that school by the total number of students in the university. Then, the number of samples allocated to each school was categorized based on the number of the male and female students in each school, alongside with their respective academic year. In turn, some classrooms in each school were selected randomly with the assistance of the random number table. Finally, in each classroom, all of the volunteer students who were present on the day of the research team's visit were invited to complete the self‐administered instruments. The survey was conducted by a trained reproductive health researcher. The sample size was determined to be 970 subjects with a 95% confidence level (*α* = 0.05), 90% power (*β* = 0.01) and 0.13 correlation coefficient between mental health and high‐risk sexual behaviour of a former study (Rosario et al., 2006) and with the assistance of G Power software. To be eligible for participation, students had to meet the following inclusion criteria: studying at the undergraduate level at the time of the survey and being in the age range of 18–25 years old. No exclusion criteria were applied.

### 2.2 Study instruments

The original version of the Youth Risk Behavior Surveillance System questionnaire was developed in 1990 by the Centers for Disease Control and Prevention to monitor priority health risk behaviours that contribute markedly to the leading causes of death, disability and social problems among youth and adults in the United States (Kann, 2016). This self‐report questionnaire contains 84 questions in six domains, including intentional and unintentional injuries (7 and 12 questions, respectively), tobacco use (12 questions), alcohol and drug use (25 questions), high‐risk sexual behaviours (8 questions), unhealthy dietary behaviours (12 questions) and physical inactivity (8 questions). The psychometric properties of the Persian version of this questionnaire show that this instrument is a valid and reliable tool for measuring risky behaviours of Iranian young people. Cronbach's *α* of the Persian version of this questionnaire in different domains ranged from 0.73–0.86. Also, its consistency was established with test–retest reliability and intracluster correlation coefficient (ICC) for subscales of intentional and unintentional injuries, tobacco use, alcohol and other drug use, high‐risk sexual behaviours, body weight and dietary behaviours and physical activity were 0.83, 0.73, 0.71, 0.64, 0.85 and 0.82, respectively, *p* < .001, with an interval of 2 weeks (Baheiraei, Hamzehgardeshi, Mohammadi, Nedjat, & Mohammadi, 2012).

A student was categorized as an individual with high‐risk behaviour (in each domain) if her/his response was positive to at least one question in each domain indicating risky behaviour according to the criteria established by the Centers for Disease Control and Prevention (Kann, 2016). For example, students were considered high‐risk if he/she had at least one or more involvement in unintentional and intentional injury behaviours (during the past 30 days before the survey), had ever tried cigarette smoking (even one or two puffs), had at least one drink of alcohol even once in a lifetime (i.e. ever drank alcohol), had used illegal drugs even once in a lifetime, had sexual intercourse with four or more persons during their lifetime or had any kind of high‐risk sexual behaviours during their last intercourse. Unhealthy dietary behaviours were defined as if did not consume milk, vegetables, fruit or 100% fruit juices or had drunk a can, bottle or glass of soda or pop (not counting diet soda or diet pop) one or more times per day during the 7 days before the survey. Insufficient physical activity was defined as not participating in at least 60 min of physical activity on at least one day during the 7 days before the study (Baheiraei, Hamzehgardeshi, Mohammadi, Nedjat, & Mohammadi, 2013; Baheiraei, Khoori, et al., 2013).

For measuring health concerns of Medical Sciences' students, Adolescent Health Concerns Inventory that was developed by Weiler in 1990 was used (Weiler, Sliepcevich, & Sarvela, 1993). This inventory contains 150 items and 12 subscales regarding concerns about: substance use and abuse, such as use of alcohol, cigarettes, diet pills (15 items); diseases and disorders such as diabetes, cancer, sleep problems (17 items); the environment such air pollution, water pollution, littering (11 items); health services such as the cost of medical care, health advertising, healthcare confidentiality (7 items), human sexuality such as close and intimate relationship with the opposite sex, talking about sex (11 items); personal health such as acne, attractiveness to others, body shape (13 items); nutrition such as eating a balanced diet, eating fast foods (8 items); injury prevention and control such as auto accidents, preventing sport injuries (13 items); social health such as discrimination, military conflict, unemployment (16 items); relationships such as falling in love, getting married, having a role model (14 items); emotional health such as dying, having faith in a religion, loneliness (17 items); and concern about the future such as being successful, getting good grade (8 items). Each item is scored dichotomously as either zero or one, and the overall score ranges from 0–150. Higher scores indicate more health concerns. The internal consistency of the Persian version of the Adolescent Health Concerns Inventory, as measured by Cronbach's *α*, ranged between 0.68–0.89 for 12 subscales and 0.96 for the total inventory. The Kappa coefficients of all items were between 0.40–0.75 or above 0.75 which indicated good to excellent agreementions of the inventory (Baheiraei, Hamzehgardeshi, et al., 2013; Baheiraei, Khoori, et al., 2013).

The reliability and the consistency of these instruments in the present project were assessed with participation of 20 university students. In this way, Cronbach's *α* 0.76 and CCI 0.88, (*p* < .001), were established for the Youth Risk Behavior Surveillance System questionnaire and Cronbach's *α* 0.94 ICC 0.74, (*p* < .001), for the Adolescent Health Concerns Inventory as well.

### 2.3 Data analysis

Descriptive findings were reported using proportions and means with standard deviations. To identify whether high‐risk behaviours (as dependent variables) are more frequent in Medical Sciences' students who experienced health concerns, we first conducted the bivariate analysis using the chi‐square test and independent *t* test. Then, a multivariate logistic regression model was administrated with the variables that were related to the high‐risk behaviours. Statistical significance was based on a *p* < .05. Analyses were performed using Statistical Package for Social Sciences for Windows version 16.0 (SPSS Inc.).

## 3 RESULTS

Of the 970 completed questionnaires, 44 were eliminated due to incomplete and inadequate replies to the questions (response rate = 95%). Among the respondents, 56.2% were female, and the mean age of the participants was 19.19 ± 1.97 years. Most of the participants (52.1%) were studying for Bachelor's degree in Science. Most of them were single (92.1%), and 45.9% lived in dormitories. The socio‐demographic characteristics of the participants are shown in Table 1.

**TABLE 1:** Socio‐demographic characteristics of Medical Science students (*n* = 926)

Variables	*N* (%)
Gender	
Female	520 (56.2)
Male	406 (43.8)
Living with both parents	
Yes	858 (92.7)
No	68(7.3)
Father's education	
Illiterate/elementary school	111 (12.4)
Secondary school/high school	325 (35.1)
University	486 (52.5)
Father's occupation	
Self‐employed	378 (40.8)
Employed	517 (55.8)
Unemployed	31 (3.4)
Mother's education	
Illiterate/elementary school	175 (18.9)
Secondary school/high school	381 (41.1)
University	370 (40.0)
Mother's occupation	
Housewife	615 (66.4)
Employed	311 (33.6)
Parents' marital status	
Living with each other	856 (92.4)
Divorced	33 (3.6)
One of the parents has died	37 (4.0)

**FIGURE 1:**
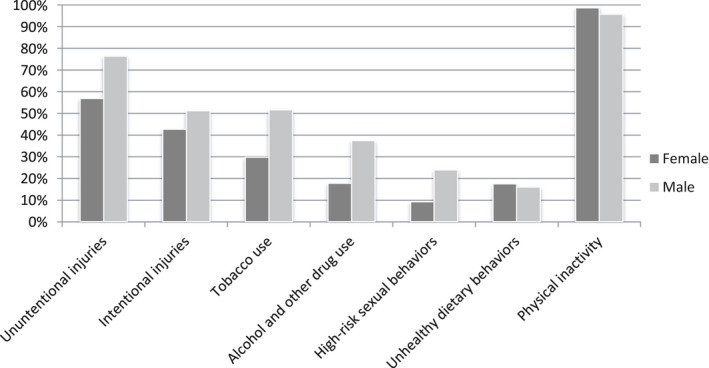
Prevalence of high‐risk behaviours by gender in Medical Science students (*n* = 926)

The results indicated that physical inactivity was the most common high‐risk behaviour that the students engaged in (97.5%, CI 95%: 96.5%, 98.5%), followed by unintentional injuries (65.4%, CI 95%:62.45%, 68.4%) and intentional injuries (46.4%, CI 95%: 43.2%, 49.6%). Intentional and unintentional injuries, smoking and tobacco use, alcohol and drug use and high‐risk sexual behaviours more frequently occurred among males (*p* < .05), whereas physical inactivity was reported more among females (Figure 1). According to Table 2, the mean and standard deviation of the total score for the students' health concerns was 35.64 ± 24.66 (CI 95%: 34.05, 37.23). The mean and standard deviation of the subscales of Adolescent Health Concerns Inventory showed that the maximum and minimum mean score related to concern about the future (4.39 ± 4.04; CI 95%: 4.04, 4.39) and health services(1.60 ± 1.77; CI 95%: 1.50, 1.72), respectively.

**TABLE 2:** Mean and standard deviation score of students' health concerns (*n* = 926)

Health Concerns	Mean	Standard deviation	Confidence Interval %95		Minimum	Maximum
			Lower limit	Upper limit		
Personal Health	4.13	2.79	3.95	4.31	0	13
Health services	1.6	1.06	1.5	1.71	0	7
The Environment	3.28	2.96	3.09	3.47	0	12
Disease and Disorders	3.64	3.34	3.19	3.67	0	17
Substance Use and Abuse	1.71	3.09	1.51	1.91	0	15
Human Sexuality	1.79	2.79	1.63	1.95	0	11
Injury Prevention and Control	2.55	2.44	2.37	2.73	0	13
Nutrition	2.09	1.96	1.96	2.21	0	7
Social Health	2.97	2.47	2.76	3.19	0	16
Relationships	3.77	3.74	3.53	4.01	0	14
Emotional Health	4.17	4.11	3.84	4.38	0	17
The future	4.21	2.64	4.04	4.39	0	8
Total Health Concern	35.64	24.6	34.05	37.23	0	150

In bivariate analysis as showed in Table 3, by considering potential covariates such as participants' age and gender and their parents' education and occupation status, university students' concerns increased the odds of risky behaviours (crude odds ratio ranged between 0.89–1.25, *p* < .05). As the goal in any data analysis is to extract from raw information the accurate estimation (Alexopoulos, 2010), we then applied a multivariate logistic regression model to calculate adjusted odds ratio (AOR). Results showed the odds of high‐risk sexual behaviours and unintentional injuries increased as students' concerns about human sexuality increased (AOR 1.39; 95% CI: 1.22, 1.57 and AOR 1.10; 95% CI: 1.01, 1.19, respectively). The participants' concerns about emotional health increased the odds of intentional injuries (AOR 1.08; 95% CI: 1.02, 1.15). The odds ratio of tobacco use increased as students' concerns about injury prevention and control increased (AOR 1.12; 95% CI: 1.01, 1.20). Moreover, when the students' concerns about nutrition increased, the odds of unhealthy dietary behaviours increased (AOR 1.13; 95% CI: 1.02, 1.26); however, an increase in worries about the future was related to lower unhealthy dietary behaviours (AOR 0.86; 95% CI: 0.70–0.84). Finally, the odds of high‐risk sexual behaviours decreased as students' concerns about diseases and disorders increased (AOR 0.89; 95% CI: 0.79, 0.98) (Table 4). In this study, no association was found between high‐risk behaviours and other health concerns such as substance use and abuse, the environment, health services, personal health, social health and relationships (*p* > .05).

**TABLE 3:** Odds ratio (Confidence Interval 95%) of correlates of high‐risk behaviours by univariate logistic regression

	Unintentional injuries	intentional injuries	Tobacco use	Alcohol and other drug use	High‐risk sexual behaviour	Unhealthy dietary behaviour	Physical inactivity
Health Concerns							
Human Sexuality	1.14***(1.07–1.21)				1.25***(1.18–1.33)		
Injury Prevention and Control			1.06*(1.01–1.11)				—
Emotional Health		1.11***(1.07–1.15)		—			
Disease and Disorder					1.00(0.96–1.05)		
Nutrition						1.07(0.98–1.16)	
The Future						0.86***(0.82–0.94)	
Gender							
Women	Ref.	—	Ref.	Ref.	—	—	—
Men	2.44***(1.83–3.25)		2.52***(1.92–3.30)	2.78***(2.05–3.76)			
Age	—	—	—	—	1.44***(1.31–1.58(	—	—
Father's education							
Illiterate/elementary school	Ref.						
Secondary school	0.60*(0.36–0.99)	—	—	—	—	—	—
University	0.52*(0.28–0.74)						
Mother's occupation							
Housewife	—	Ref.	—	—	—	—	—
Employed		1.49**(1.13–1.96)					
Father's occupation							
Self‐employed				Ref.	Ref.		
Employed	—	—	—	0.91(0.67–1.23)	0.57**(0.39–0.82)	—	—
Unemployed				2.96**(1.41–6.21)	1.42(0.61–3.32)		
Parents' marital status							
Live with each other				Ref.			
Divorced	—	—	—	5.31***(2.57–10.98)	—	—	—
One of the parents has died				1.28(0.62–2.64)			

* *p*‐value < .05.

** *p*‐value < .01.

*** *p*‐value < .001.

^†^ As there is no significant relationship between the some variables in above table (*p*‐value > .05), their cells are blank.

**TABLE 4:** Adjusted odds ratio (confidence interval 95%) of correlates of high‐risk behaviours by multivariate logistic regression

	Unintentional injuries	intentional injuries	Tobacco use	Alcohol and other drug use	High‐risk sexual behaviour	Unhealthy dietary behaviour	Physical inactivity
Health Concerns							
Human Sexuality	1.10* (1.01–1.19)				1.39***(1.22–1.57)		
Injury Prevention and Control			1.12*(1.01–1.20)				
Emotional Health		1.08**(1.02–1.15)		—	0.89*(0.79–0.98)		—
Disease and Disorder							
Nutrition						1.13*(1.02–1.26)	
The Future						0.86**(0.79–0.94)	
Gender							
Women	Ref.	—	Ref.	Ref.	—	—	—
Men	2.12***(1.60–3.04)		1.62***(1.15–2.25)	1.76**(1.16–2.72)			
Age	—	—	—	—	1.23*(1.01–1.50)	—	—
Father's education							
Illiterate/elementary school	Ref.						
Secondary school	00.77 (0.42–1.40)	—	—	—	—	—	—
University	0.52*(0.26–0.92)						
Mother's occupation							
Housewife	—	Ref.	—	—	—	—	—
Employed		1.53*(1.09–2.15)					
Father's occupation							
Self‐employed				Ref.	Ref.		
Employed	—	—	—	1.26(0.82–1.95)	0.52*(0.30–0.90)	—	—
Unemployed				3.88*(1.10–13.62)	0.20*(0.46–0.87)		
Parents' marital status							
Live with each other				Ref.			
Divorced	—	—	—	14.39**(2.23–9.86)	—	—	—
One of the parents has died				2.13 (0.23–19.44)			

* *p*‐value < .05.

** *p*‐value < .01.

*** *p*‐value < .001.

^†^ As there is no significant relationship between the some variables in above table (*p*‐value > .05), their cells are blank.

## 4 DISCUSSION

University students are important targets for the promotion of healthy behaviours of the adult population. In this study, high‐risk behaviours such as intentional and unintentional injuries, alcohol use and high‐risk sexual behaviours were higher in male students. Many studies in line with our study showed that high‐risk behaviours are gender‐related and multiple risk‐taking behaviours more common in men (Al‐Harbi & Farajat, 2019; Ansari et al., 2011; Kritsotakis, Psarrou, Vassilaki, Androulaki, & Philalithis, 2016). The prevalence of insufficient physical activity in the present study, in addition to being much higher than in the Western and Middle Eastern countries, was more frequent in female students (Ansari et al., 2016; Deyab & Abdelrahim, 2019; Fessikh et al., 2018; Grasdalsmoen et al., 2019; Sorush et al., 2018). This could be due to the some limited facilities and cultural barriers in Middle‐east countries like Iranian context for women to exercise, jogging or cycling in public (Al‐Harbi & Farajat, 2019). The most concern of the participants was related to worry about the future. In accordance with the present study, it showed that worry about being successful, choosing a job, getting a good grade, the future in the years to come was the most concerns of the young people (Baheiraei et al., 2016; Firouzkouhi et al., 2006).

Our finding about the relationship between concern about human sexuality (e.g. concerns about having sexual intercourse, physical sexual development and pressure to have sex) and high‐risk sexual behaviours is in line with the other studies (Maldonado et al., 2015; Teychenne et al., 2015). For example, it showed that individuals who were more concerned about sexual attraction in relation to opposite sex reported more engagement in sexual risky behaviours like unprotected sex and the use of alcohol and drugs before sexual intercourse (Auslander, Baker, & Short, 2012; Merianos, King, & Vidourek, 2013). It seems an increase in concerns about relationships with the opposite sex may reduce people's self‐confidence regarding safe sexual behaviour. This issue discouraging them from strongly insisting on preventive measures such as protected sex and the use of condoms and other contraceptive methods (Bi, Ma, Yuan, & Zhang, 2016). In contrast, it is showed that people who were more concerned with body image and appearance had less confidence in establishing relationships, which in turn resulted in the occurrence of fewer high‐risk sexual behaviours (Littleton, Axsom, & Pury, 2005).

The more intentional injury in the university students who have concerns about emotional health may indicate such students seek relief from the consequent stress and tension by resorting to risky behaviours (Palti, Halevy et al., 1995; Tavolacci et al., 2013). Also, association between university students' concerns about injury prevention and control (such as being murdered and driving accidents) with substance use indicated that both improving safe environment and reducing the negative psychological effects of unsafe conditions are strongly needed (Smalley et al., 2017). Also, the predisposing role of students' concerns about the future (such as concern in relation to academic and professional achievements) to unhealthy dietary behaviours may suggested that intense feeling of insecurity about their financial and occupational rehabilitation, manifested by symptoms such as eating disorders (Eisenberg et al., 2007; Fradelos et al., 2019). Due to limited studies on the relationship between health concerns and high‐risk behaviours in medical students, it was not possible to compare some findings of the present study with the other studies. The more high‐risk sexual behaviours in university students with the more concerns about diseases and disorders (such as concern about chronic diseases and sexually transmitted diseases) are an example. Although it seems diseases can affect people not only physically, but also mentally, and finally, can alter the affected person's perspective on life and involving in risky behaviours.

In the present study, Medical Sciences' students who had more concerns about nutrition were more likely to exhibit unhealthy dietary behaviours. A study on Spanish students showed the importance of body weight and fitness and being female were some of the significant predictors of unhealthy methods employed to lose weight (Blow, Taylor, Cooper, & Redfearn, 2010). Also, it is indicated that with increasing concerns about self‐esteem, eating disorders and unhealthy nutritional behaviours generally will be increased (Sassaroli & Ruggiero, 2005). Moreover, some studies reported that weight‐related concerns might relate to the onset of smoking and tobacco use (Harakeh, Engels, Monshouwer, & Hanssen, 2010; Palti et al., 1995). However, on the contrary, it is reported that students who were concerned about nutrition and calorie intake may exhibited fewer high‐risk dietary behaviours (Sun, 2008). The results of the present study may indicate that concerns about certain health issues can have an important role in preventing some high‐risk behaviours. For example, concern about disease and disorders was associated with reduced high‐risk sexual behaviours, and the students who were concerned about the future were less likely to have unhealthy dietary behaviours. These findings may be evidence that high‐risk behaviour may decrease as knowledge and worries increase (Arroyo et al., 2004; Yahia et al., 2008).

## 5 STRENGTHS AND LIMITATIONS

As entering to the Iranian university is followed by a national entrance examination (named KOONKOOR) so the participants of the present study in MAZUMS were students from all over the country and from different ethnic groups. This could increase the generalizability of the findings as the results would be more acceptable and representative if the samples are taken from diverse localities with students of diverse backgrounds, although the results may not be generalized to non‐medical sciences' students. Comparing the gender composition of the students in the present study (more than 56% of students were female) with the national statistics (Iran Statistical Center, 2018), which shows the highest number of female students in medical universities are female for different reasons for example Midwifery education in Iranian universities belongs to girls, merely), can be indicated that the study population is a representative of the students in the whole country and the probability of the selection bias is minimal.

However, our results must be interpreted in the light of some limitations that are worth discussing. First, because this study was cross‐sectional, determining the cause‐and‐effect relationships between health concerns and high‐risk behaviours is impossible due to the temporality bias. Second, information bias is possible due to the sensitive nature of the questions, especially in the context of conservative societies such as the Islamic Republic of Iran, where both cultural and religious restrictions prevent students from revealing their darkest secrets. To reduce this bias, although we reassure students of the confidentiality of the data, nevertheless, students may still have been reluctant to answer these questions honestly and the results may be under‐estimated and the results may be prone to social desirability bias. Third, the scope of the research was limited as other factors that may affect engagement in high‐risk behaviours (e.g. type of personality and childhood experiences) were not investigated. Moreover, the results of this research are only generalizable to Medical Science students. Fourth, there is a possibility of interview bias even though the interviewer attempted to read from the questionnaire when asking the question and avoid facial expressions of change voice and notations during the interview. Confounding bias was controlled by using the regression analysis. Final limitation of this study refers to the sampling method. In the final step of sampling, all of the volunteer students who were present on the day of the research team's visit were invited to complete the self‐administered instruments. This not‐randomly participation of the students may have increased the likelihood of selection bias, but this was inevitable as if we asked students to answer these culturally sensitive questionnaires according to the randomized selection in each class, it might increase the information bias, because they might be wondering why I was chosen to answer? However, based on the randomly selection of the classrooms in each strata, the students have an equal chance of being included and the selection bias can be limited.

## 6 CONCLUSION

Medical Sciences' students serve as role models for the other students and the transition from being a university student to being a good healthcare practitioner (like a good: doctor; nurse; midwife) requires committed role models. So, the findings of this project by investigating the university students' concerns in predisposing them to risky behaviours have some implication in practice and policy. This study has shown various prevalence rates of risky behaviours and health concerns among Medical Sciences' students at MAZUMS; hence, university‐based health education and promotion programs are required. Also, these findings illustrate the need to consider health concerns as predisposing variables when investigating the high‐risk behaviours of young people. Our study points to the necessity for screening, prevention and interventional programs targeting mental health in university students Further studies are recommended to reach an in‐depth understanding of how Iranian university students feel towards life in general, how often they feel concerned and how intense their concerns are. It is demonstrated that there is a large discrepancy in the literature about the prevalence and related factors of some high‐risk behaviours such as dietary behaviours and physical activity. This issue may be resulted from that there is not an acceptable national standards to facilitate comparison between countrywide studies. Also, it is not entirely clear to what extent high‐risk behaviours and health concerns can seriously endanger the health of young people? So, it is important to develop the culture‐based instruments for measuring high‐risk behaviours and health concerns with a qualitative approach and through in‐depth interviews with university students in the future. In this way, the roles of new emerging social determinants of health such as social media are recommended to be in the spotlight.

## CONFLICT OF INTEREST

The authors declare that they have no conflict of interest.

## AUTHOR CONTRIBUTIONS

All authors made a substantial contribution to writing of the paper draft and met the four criteria for authorship recommended by the International Committee of Medical Journal Editors.

## ETHICAL APPROVAL

All procedures performed in the studies involving human participants were in accordance with the ethical standards of the institutional and/or National Research Committee and with the 1964 Helsinki declaration and its later amendments or comparable ethical standards. The Ethics Committee of MAZUMS approved the study (Ethical code: IRMAZU MS.REC. 95.2 446), and the students were informed about the topic, purpose and procedure of the study. All volunteer students were provided informed written consent before participation and were assured of the confidentiality of the data. In this way, a covered box was designed to collect anonymous questionnaires and reassure students that the information was confidential.

## Data Availability

Data that support the findings of this study are available from the corresponding author upon request.
